# Inducing Intermediates in Biotransformation of Natural Polyacetylene and A Novel Spiro-γ-Lactone from Red Ginseng by Solid Co-Culture of Two Gut *Chaetomium globosum* and The Potential Bioactivity Modification by Oxidative Metabolism

**DOI:** 10.3390/molecules25051216

**Published:** 2020-03-08

**Authors:** Bang-Yan Wang, Chen-Hao Zhu, Xue-Qiong Yang, Ming Hu, Ting-Ting Xu, Xue-Yin Wang, Shuang Yang, Ya-Bin Yang, Zhong-Tao Ding

**Affiliations:** Functional Molecules Analysis and Biotransformation Key Laboratory of Universities in Yunnan Province, Key Laboratory of Medicinal Chemistry for Natural Resource, Ministry of Education, School of Chemical Science and Technology, Yunnan University, 2st Cuihu North Road, Kunming 650091, China; gjk201113409218@163.com (B.-Y.W.); zhuchenhaots@163.com (C.-H.Z.); yangxq@ynu.edu.cn (X.-Q.Y.); Hm1720553468@ynu.edu.cn (M.H.); TIXUTing@163.com (T.-T.X.); jinggui_nxdx@163.com (X.-Y.W.); ys19950814@163.com (S.Y.)

**Keywords:** fermented red ginseng, *Chaetomium globosum*, polyacetylene, detoxification, oxidative metabolism

## Abstract

The ω-hydroxyl-panaxytriol (**1**) and ω-hydroxyl-dihydropanaxytriol (**2**)—are rare examples of polyacetylene metabolism by microbial transformation, and these new metabolites (**1**, **2**) from fermented red ginseng (FRG) by solid co-culture induction of two *Chaetomium globosum* should be the intermediates of biotransformation of panaxylactone (metabolite **A**). The metabolic pathway of panaxylactone was also exhibited. The ingredients of red ginseng (RG) also induced the production of rare 6/5/5 tricyclic ring spiro-γ-lactone skeleton (**3**). The ω-hydroxylation of new intermediates (**1**, **2**) decreases cytotoxicity and antifungal activity against *C. globosum* compared with that of its bioprecursor panaxytriol. Additionally, compounds **1** and **2** indicated obvious inhibition against nitric oxide (NO) production, with ratios of 44.80 ± 1.37 and 23.10 ± 1.00% at 50 μM. **1** has an equivalent inhibition of NO production compared with the positive drug. So, the microbial biotransformation that occurred in FRG fermented by gut *C. globosum* can change the original bioactivity of polyacetylene, which gave a basis about the metabolic modification of red ginseng by intestinal fungus fermentation.

## 1. Introduction

**Figure 1:**
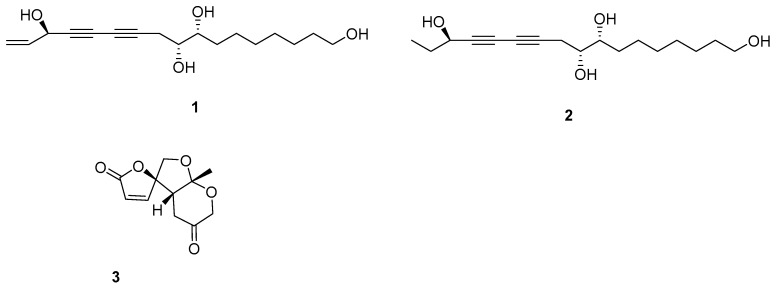
The structures of isolated compounds.

As one of the most popular herbal medicines, red ginseng (RG) has been studied for its pharmacological activities, and it is also a widely used functional food [1]. The gut microbiota has an application for medical treatment and drug discovery over distinct pathema indications [2]. It has been reported that ginseng was processed by fungal and bacterial fermentation, which can cause an increase in the bioactivity and biotransform ginsenosides into aglycone forms [3,4]. The fermentation process had the potential for biotransformation of active metabolites into a high-value product, and it can also contribute to the improvement of biological activities of the medicinal plant [5]. Co-culture technology has appeared as a new approach to activating the biosynthesis of natural products [6]. The natural world was the co-culture system, not single cultures, so the co-culture system can imitate the interaction between different strains. *Chaetomium globosum*, belonging to the *Chaetomiaceae* family, is commonly found in terrestrial and marine habitats, and it was reported to cause infections in humans [7]. *C. globosum* was also found residing in *Epinephelus drummondhayi* guts [8]. On the positive side, the *Chaetomium* genus afforded a rich source of unique bioactive metabolites [9]. The emergence of antibiotic-resistant pathogenic microorganisms poses a threat to human health [10]. Most of the drug resistance was about the chemicals; the natural medicine was not studied in-depth study. In this work, a pathogen resistance against natural medicine was found by chance. Our previous work led to a rare example of polyacetylene biotransformation and a new polyacetylene, panaxylactone (metabolite **A**) transformed from panaxytriol in fermented red ginseng (FRG) by process of *C. globosum* [11]. The biotransformation process was not clear, so the reinvestigation of the polyacetylenes from FRG by solid co-culture of two *C. globosum* from different sources was carried out. Two new intermediates (**1**, **2**) relevant to the biotransformation of panaxylactone were isolated, along with a microbial metabolite having a novel spiro-γ-lactone skeleton (chaetoginsin, **3**) (Figure 1). The metabolic modification of active polyacetylenes from RG was investigated by these compounds from FRG by pathogen *C. globosum*.

## 2. Results and Discussion

**Figure 2:**
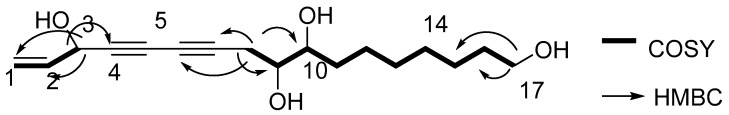
Correlation spectroscopy (COSY) correlations and heteronuclear multiple bond correlation (HMBC) correlations of new compound **1.**

Compound **1** was gained as an oil and the formula was identified to be C_17_H_26_O_4_ by its Distortionless Enhancement by Polarization Transfer (DEPT) spectrum and HR-ESI-MS (m/z 317.1735 [M + Na]^+^). Analysis of its ^13^C-NMR data exhibited the presence of the panaxytriol skeleton. The analysis of correlation spectroscopy (COSY) spectroscopic data revealed the C_8_-C_9_-C_10_-C_11_-C_12_-C_13_-C_14_-C_15_-C_16_-C_17_ structural connection. The heteronuclear multiple bond correlation (HMBC) correlations from H-3 to C-1, C-2, and C-4; H-8 to C-6, C-7, C-9 and C-10; H-17 to C-15 and C-16 elucidate the structure of compound **1** (Figure 2). There is a high similarity between the chemical skeletons of compound **1** and known compound panaxytriol (PXT) [12,13]; therefore, a polyacetylene with cytotoxicity previously isolated in ginseng, the configuration of compound **1** at C-3 was identified by comparing the NMR data and the optical rotation (OR) values with those of panaxytriol and metabolite **A** found from FRG in our previous work [11]. The absolute configuration of **1** was confirmed by comparing OR value at −24.0 and circular dichroism (CD) spectrum with those of known panaxytriol [11,12]. The structure of compound **2** was determined as the dihydrogen at C-1, C-2 derived from **1** by DEPT spectrum, HRESI-MS, 2D-NMR, optical rotation value, and CD spectrum. 

**Figure 3:**
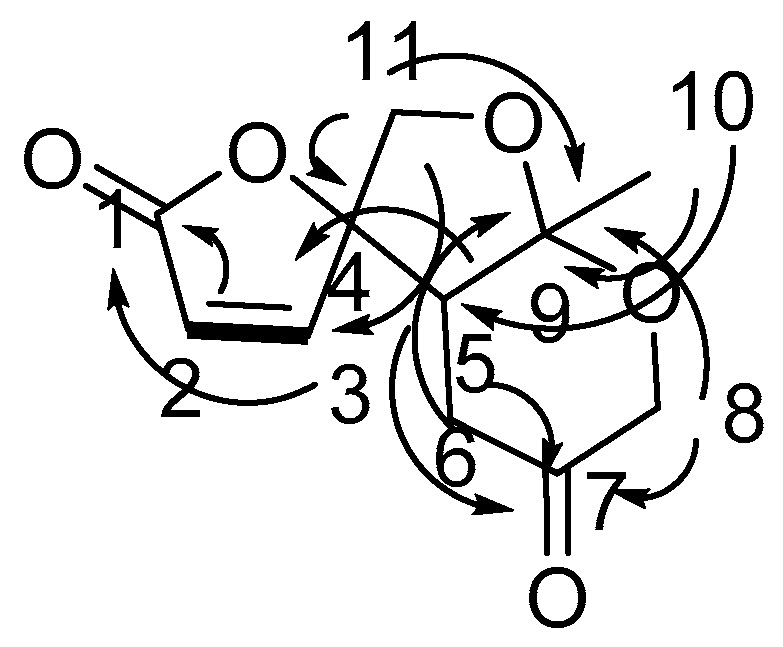
COSY correlations and HMBC correlations of chaetoginsin (**3**).

Compound **3** was determined to be C_11_H_12_O_5_ from its NMR spectrum and HR-ESI-MS. Analysis of its DEPT data exhibited one lactone carbonyl (δ_C_ 168.7), one ketone (δ_C_ 204.9), and two olefinic carbons (δ_C_ 124.1 and 152.3), so **3** has a tricyclic ring system. The structure of **3** (Figure 3) was confirmed by HMBC spectrum from H-10 to C-5, C-9; from H-8, H-6,H-11 to C-9; from H-11 to C-4; from H-5, H-6, H-8 to C-7; from H-5, H-11 to C-3; from H-2, H-3 to C-1. The relative configurations of **3** at H-5 and H-9 were determined by the nuclear overhauser enhancement spectroscopy (ROESY) correlations between H-5 and H-10. The absolute configuration of **3** was determined by comparing the circular dichroism spectrum with that of its analog, streptoglyceride A [14].

**Figure 4:**
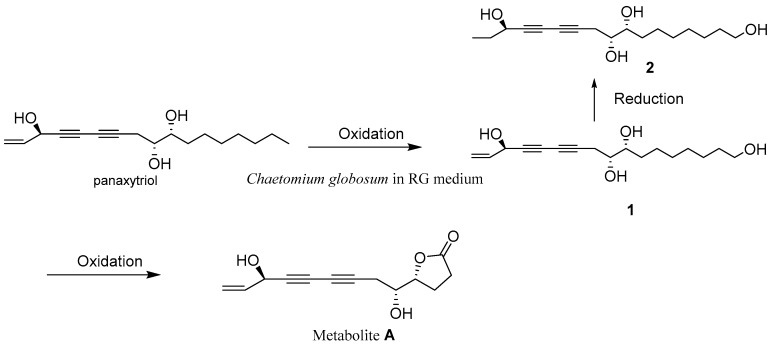
The plausible biotransformation of panaxylactone (metabolite **A**).

In our previous work, some active compounds such as panaxytriol from RG indicated inhibition against the merisis of *C. globosum*, and *C. globosum* may own a detoxication mechanism against the antifungal activity of active compounds [11]. Panaxylactone (metabolite **A**) biotransformed from panaxytriol exhibited no antifungal activity, suggesting that *C. globosum* can transform panaxytriol to panaxylactone with less antifungus activity than can panaxytriol for its own merits. The biotransformation of panaxylactone was not clear, so the investigation of the intermediates in the biotransformation of natural polyacetylene by adjustment of fermentation mode was carried out. Two new key intermediates (**1**, and **2)** were isolated, and the metabolism process of panaxylactone was shown in Figure 4. The natural 1-hydroxydihydropanaxacol from ginseng like the structure of **2,** except for one more ω-hydroxy at **2**, had *S* configuration at C-3 [15], and **2** had *R* configuration at C-3, so **2** was mostly transformed from **1**. Compound **1**, **2,** and **3** also exhibited no antifungal activity with MICs > 256 μg/mL against *C. globosum*, which gave more proof on the detoxication mechanism of *C. globosum* against the antifungal activity of polyacetylenes from RG. Numerous bioactive polyacetylenes were found in the plant *Panax ginseng*. Among these compounds, PXT was shown in vitro cytotoxic activity against a series of human tumor cells [13]. Compound **1**, **2** indicated no cytotoxicity with a concentration of 40 μM. Based on the LC-MS test (Figure S25), the new compound **2** was exhibited to be non-mark in RG extract, but mark in FRG, so compound **2** was obtained by microbial fermentation. Through these results, it was found that the changes in the chemical structure of PXT can decrease the antifungal activity and cytotoxicity. Compounds **1** and **2** indicated significant inhibition against NO production, with ratios of 44.80 ± 1.37 and 23.10 ± 1.00% at 50 μM. So, pathogen *C. globosum* might possess the potential of bioactivity modification upon the polyacetylenes from RG. The novel skeleton, like **3,** was only found in basidiomycete *Lenzites betulinusrare* and *Streptomyces*, but not from plants [14,16]. **3** was positive in FRG by LC-MS, but negative in RG and liquid medium of *C. globosum*, so **3** should be produced by *C. globosum,* and this skeleton was first found in the generous *Chaetomium* metabolites. Thus, the chemical constituents of RG extract can induce the production of rare 6/5/5 tricyclic ring spiro-γ-lactone skeleton (**3**) from *Chaetomium*.

## 3. Materials and Methods 

### 3.1. General Experimental Procedure 

Silica gel (200–300 mesh), Lichroprep RP-18, and Sephadex LH-20 were applied to column chromatography. The TLC plate was purchased from Qingdao Marine Chemical Group Co. and used for chromatography analysis. One-dimensional and two-dimensional NMR data were acquired on a Bruker AVANCE-600 MHz NMR instrument (Bruker, Karlsruhe, Germany). Mass spectra were measured by spectrometers of Agilent G3250AA (Agilent, Santa Clara, CA, USA) and AutoSpec Premier P776 (Waters, Milford, MA, USA). Optical rotations and circular dichroism spectra were measured on a polarimeter (Jasco P-1020) and an Applied Photophysics Chirascan spectrometer (Applied Photophysics Ltd., Surrey, British). LC-MS analyses were performed on an instrument (LTQ ORBITRAP XL). 

### 3.2. Fungus Material and Cultivation Condition

Red ginseng was purchased from the Juhuacuen medicinal market of Kunming in Yunnan Province in China. The fungi were obtained from *Dendrobium officinale* gathered in Honghe of Yunnan Province and *Hydrocharis dubia* collected at Lijiang in Yunnan Province, China. The species were determined as *C. globosum* (JC-H8, YNH-16) on the analysis of morphological and genetic (ITS) properties. The voucher specimens were conserved in the analytical chemistry key laboratory of universities in Yunnan Province. The fermentation of two *C. globosum* were put into effect in 500 mL Erlenmeyer flasks consisting of 0.12 L of potato dextrose broth (PDB) with potato infusion of 0.024 kg of fresh potato, 1.8 g of dextrose, 120 mL of distilled H_2_O, pH 7.0, at 150 rpm and 28 °C for three days for a seed medium. Each 20–25 mL of seed medium was put into a 0.35 L microbiological culture vessel containing 0.12 kg of cooked RG. A total of 7 kg RG was fermented by *C. globosum* at 28 °C for thirty days. 

### 3.3. Extraction and Separation Methods

All 7000 g of the solid medium was extracted with methanol for three times, the methanol extract was partitioned with ethyl acetate to give an EtOAc fraction and then dried under vacuum to obtain a dark extract (52.3 g). The extract was applied to column chromatography on silica gel (7 cm × 20 cm, 200 to 300 mesh, 400 g) and was eluted with a solvent system of chloroform-methanol from 1:0 to 10:1 (*v*/*v*) to give four fractions (A–D). Fraction A (13.3 g) was purified by using Lichroprep RP-18 (3 cm × 10 cm, CH_3_OH-H_2_O at 20% to 80%), Sephadex LH-20 (2 cm × 100 cm, chloroform-methanol at 1:1) to get **2** (3 mg). Fraction B (13.2 g) was separated by Sephadex LH-20 (2 cm × 150 cm, chloroform-methanol at 1:1), Lichroprep RP-18column chromatography (3 cm × 12 cm, CH_3_OH-H_2_O at 10% to 80%) to get **1** (4 mg), and chaetoginsin (**3**, 6 mg). 

ω-Hydroxyl-panaxytriol (**1**). [α${]}_{D}^{22}$ −24.0 (c 0.35, MeOH). HR-ESIMS *m/z*: 317.1735 [M + Na]^+^, calcd for C_17_H_26_O_4_Na: 317.1729. ^1^H-NMR (MeOD, 600 MHz) and ^13^C-NMR (MeOD, 150 MHz) in Table 1. 

ω-Hydroxyl-dihydropanaxytriol (**2**). [α${]}_{D}^{22}$ −8.6 (c 0.45, MeOH). HR-ESIMS *m/z*: 319.1879 [M + Na]^+^, calcd for C_1__7_H_2__8_O_4_Na: 319.1885. ^1^H-NMR (MeOD, 600 MHz) and ^13^C-NMR (MeOD, 150 MHz) in Table 1. 

Chaetoginsin (**3**). [α${]}_{D}^{22}$ −21.6 (c 0.14, MeOH). HR-ESIMS *m/z*: 247.0578 [M + Na]^+^, calcd for C_11_H_12_O_5_Na: 247.0582. ^1^H-NMR (CDCl_3_, 600 MHz) and ^13^C-NMR (CDCl_3_, 150 MHz) in Table 1. 

### 3.4. LC-MS Method

The extracts of RG, and FRG fermentation materials were afforded according to the preparation method exhibited above. Similar sampling volumes of RG, and FRG fermentation materials, and compounds **2**, **3** were prepared for application in the LC-MS investigation. A gradient solvent (methanol/0.1% methanoic acid from 40% to 99%) and a velocity of flow at 0.3 mL/min were applied to the investigation. The target ions were extracted by the precise molecular weight of compounds **2** and **3**.

### 3.5. Bioassay Method

The two-fold plate dilution method were applied to the in vitro antifungus experiment [11]. Nystatin as positive drug had a minimum inhibitory concentration (MIC) at 32 μg/mL. The method of 3-(4,5-dimethylthiazol-2-yl)-5(3-carboxymethoxyphenyl)-2-(4-sulfopheny)-2*H*-tetrazolium (MTT) assay was applied to the investigation of cytotoxicity of compounds **1** and **2** against tumor cell lines [17], A-549, HL-60, MCF-7, SW480, and SMMC-7721. Taxol was the positive drug with the IC_50_ < 0.008 μM. The NO inhibitory activities of compounds **1**,**2** were determined using the Griess reagent assay for NO production [11]. The positive control, L-NMMA has inhibitory ratio of 53.52 ± 0.53 % at concentration of 50 μM.

## Figures and Tables

**Table 1:** ^13^C Nuclear Magnetic Resonance (NMR) and ^1^H-NMR data of compounds **1**–**3.**

Pos.	1		2		Chaetoginsin	
	*δ*_H_	*δ*_C_	*δ*_H_	*δ*_C_	*δ*_H_	*δ*_C_
1	5.20 (d, *J* = 12.0 Hz) 5.42 (d, *J* = 18.0 Hz)	115.1	1.00 (t, *J* = 7.2 Hz)	8.4		168.7
2	5.93 (m)	136.9	1.68 (m)	30.5	6.23 (d, *J* = 6.0 Hz)	124.1
3	4.63 (s)	62.5	4.29 (t, *J* = 6.6 Hz)	63.0	7.26 (d, *J* = 6.0 Hz)	152.3
4		74.6		76.8		94.2
5		69.7		68.5	2.83 (m)	48.5
6		65.1		64.8	2.09 (d, *J* = 18.0 Hz), 2.51 (m)	32.7
7		78.3		77.5		204.9
8	2.49, 2.61 (m)	23.5	2.49, 2.60 (m)	23.5	3.94 (d, *J* = 18.0 Hz), 4.60 (d, *J* = 18.0 Hz)	67.5
9	3.61 (m)	72.1	3.61 (m)	72.1		106.8
10	3.59 (m)	72.4	3.59 (m)	72.4	1.64 (s)	25.1
11	1.55 (m)	32.6	1.54 (m)	32.6	3.94 (d, *J* = 12.0 Hz), 4.12 (d, *J* = 10. 8 Hz)	71.8
12	1.38 (m)	25.5	1.38 (m)	25.5		
13	1.38 (m)	29.4	1.38 (m)	29.3		
14	1.38 (m)	29.3	1.38 (m)	29.1		
15	1.38 (m)	25.5	1.38 (m)	25.5		
16	1.55 (m)	32.2	1.54 (m)	32.2		
17	3.59 (m)	61.6	3.59 (m)	61.6		

## Supplementary Materials

^1^H and ^13^C-NMR, HSQC, HMBC, ^1^H-^1^H COSY, ROESY, and HR-ESIMS spectra of compounds **1**–**3**. CD spectra of **1**, **2**, and **3**. LC-HRMS finger-prints by ion extraction of co-culture, single strain, the liquid medium of *C. globosum* fermentation products, red ginseng, and compounds (**2**, **3**). Cytotoxicity of compounds **1**, **2** by the 3-(4,5-dimethylthiazol-2-yl)-5(3-carboxymethoxyphenyl)-2-(4-sulfopheny)-2*H*-tetrazolium (MTS) method.

## References

[1] Jin S., Jeon J.H., Lee S., Kang W.Y., Seong S.J., Yoon Y.R., Choi M.K., Song I.S. (2019). Detection of 13 ginsenosides (Rb1, Rb2, Rc, Rd, Re, Rf, Rg1, Rg3, Rh2, F1, compound K, 20(S)-protopanaxadiol, and 20(S)-protopanaxatriol) in human plasma and application of the analytical method to human pharma cokinetic studies following two week-repeated administration of red ginseng extract. Molecules.

[2] Zimmermann M., Zimmermann-Kogadeeva M., Wegmann R., Goodman A.L. (2019). Mapping human microbiome drug metabolism by gut bacteria and their genes. Nature.

[3] Kim S.T., Kim H.B., Lee K.H., Choi Y.R., Kim H.J., Shin S.I., Gyoung Y.S., Joo S.S. (2012). Steam-dried ginseng berry fermented with *lactobacillus plantarum* controls the increase of blood glucose and body weight in type 2 obese diabetic db/db mice. J. Agric. Food Chem..

[4] Lee N.K., Paik H.D. (2017). Bioconversion using lactic acid bacteria: Ginsenosides, GABA, and phenolic Compounds. J. Microbiol. Biotechnol..

[5] Parshikov I.A., Woodling K.A., Sutherland J.B. (2015). Biotransformations of organic compounds mediated by cultures of *Aspergillus niger*. Appl. Microbiol. Biot..

[6] Wang R.F., Zhao S.J., Wang Z.T., Koffas M.A. (2020). Recent advances in modular co-culture engineering for synthesis of natural products. Curr. Opin. Biotech..

[7] Wang X.W., Lombard L., Groenewald J.Z., Li J., Videira S.I.R., Samson R.A., Liu X.Z., Crous P.W. (2016). Phylogenetic reassessment of the *Chaetomium globosum* species complex. Persoonia.

[8] Yan W., Ge H.M., Wang G., Jiang N., Mei Y.N., Jiang R., Li S.J., Chen C.J., Jiao R.H., Xu Q. (2014). Pictet–Spengler reaction-based biosynthetic machinery in fungi. PNAS..

[9] Fatima N., Muhammad S.A., Khan I., Qazi M.A., Shahzadi I., Mumtaz A., Hashmi M.A., Khan A.K., Ismail T. (2016). *Chaetomium* endophytes: a repository of pharmacologically active metabolites. Acta Physiol. Plant..

[10] Chang Z., Yadav V., Lee S.C., Heitman J. (2019). Epigenetic mechanisms of drug resistance in fungi. Fungal Genet. Biol..

[11] Wang B.Y., Yang X.Q., Hu M., Shi L.J., Yin H.Y., Wu Y.M., Yang Y.B., Zhou H., Ding Z.T. (2019). Biotransformation of natural polyacetylene in red ginseng by *Chaetomium globosum*. J. Ginseng Res..

[12] Satoh M., Ishii M., Watanabe M., Isobe K., Uchiyama T., Fujimoto Y. (2002). Absolute structure of panaxytriol. Chem. Pharm. Bull..

[13] Gurjar M.K., Kumar V.S., Rao B.V. (1999). Synthesis of a new type of antitumour agent panaxytriol: Synthesis of its four diastereomers. Tetrahedron.

[14] Choi B.K., Park S.Y., Choi D.K., Shin B., Shin Y.H., Oh D.C., Lee H.S., Lee H.S., Lee Y.J., Lee J.S. (2018). Streptoglycerides A−D with a rare 6/5/5 tricyclic ring skeleton from a marine actinomycete *Streptomyces* species. Org. Lett..

[15] Fujimoto Y., Satoh M., Takeuchi N., Kirisawa M. (1990). Synthesis and absolute configurations of the cytotoxic polyacetylenes isolated from the callus of *Panax ginseng*. Chem. Pharm. Bull..

[16] Wen C.N., Chen H.P., Zhao Z.Z., Hu D.B., Li Z.H., Feng T., Liu J.K. (2017). Two new γ-lactones from the cultures of basidiomycete *Lenzites betulinus*. Phytochemistry Lett..

[17] Szoka L., Isidorov V., Nazaruk J., Stocki M., Siergiejczyk L. (2019). Cytotoxicity of triterpene seco-acids from *Betula pubescens* buds. Molecules.

