# Supplementary material for: Inducing Intermediates in Biotransformation of Natural Polyacetylene and A Novel Spiro-γ-Lactone from Red Ginseng by Solid Co-Culture of Two Gut Chaetomium globosum and The Potential Bioactivity Modification by Oxidative Metabolism
Source: Molecules. 2020 Mar 8;25(5):1216. doi: 10.3390/molecules25051216 (PMC7179436; doi:10.3390/molecules25051216)
Supplement: Supplementary file 1 [file molecules-25-01216-s001.pdf]

Supplementary Materials

Inducing Intermediates in Biotransformation of Natural Polyacetylene and A Novel Spiro-γ-lactone from Red Ginseng by Solid Co-culture of Two Gut *Chaetomium globosum* and The Potential Bioactivity Modification by Oxidative Metabolism

Bang-Yan Wang †, Chen-Hao Zhu †, Xue-Qiong Yang, Ming-Hu, Ting-Ting Xu, Xue-Yin Wang, Shuang Yang, Ya-Bin Yang * and Zhong-Tao Ding *

Functional Molecules Analysis and Biotransformation Key Laboratory of Universities in Yunnan Province, Key Laboratory of Medicinal Chemistry for Natural Resource, Ministry of Education, School of Chemical Science and Technology, Yunnan University, 2st Cuihu North Road, Kunming 650091, China; gjk201113409218@163.com (B.-Y.W.); zhuchenhaots@163.com (C.-H.Z.); yangxq@ynu.edu.cn (X.-Q.Y.); Hm1720553468@ynu.edu.cn (M.-H.); TIXUTing@163.com (T.-T.X.); jinggui_nxdx@163.com (X.-Y.W.); ys19950814@163.com (S. Y.)

* Correspondence: ybyang@ynu.edu.cn (Y.-B.Y.); ztding@ynu.edu.cn (Z.-T.D.)

† These authors contributed equally to this work

Table of contents

FigureS1. HRESIMS spectrum of compound **1** 3

Figure S2. ^{1}H NMR spectrum of compound **1** in MeOD (600MHz) 3

Figure S3. ^{13}C NMR spectrum of compound **1** in MeOD (150MHz) 4

Figure S4. COSY spectrum of compound **1** in MeOD (600 MHz) 4

Figure S5. HSQC spectrum of compound **1** in MeOD (600 MHz) 5

Figure S6. HMBC spectrum of compound **1** in MeOD (600 MHz) 5

Figure S7. ROESY spectrum of compound **1** in MeOD (600 MHz) 6

Figure S8. HRESIMS spectrum of compound **2** 6

Figure S9. ^{1}H NMR spectrum of compound **2** in MeOD (600MHz) 7

Figure S10. ^{13}C NMR spectrum of compound **2** in MeOD (150MHz) 7

Figure S11.COSY spectrum of compound **2** in MeOD (600 MHz) 8

Figure S12. HSQC spectrum of compound **2** in MeOD (600 MHz) 8

Figure S13. HMBC spectrum of compound **2** in MeOD (600 MHz) 9

Figure S14. ROESY spectrum of compound **2** in MeOD (600 MHz) 9

Figure S15. HRESIMS spectrum of compound **3** 10

Figure S16. ^{1}H NMR spectrum of compound **3** in $CDCl_3$ (600MHz) 10

Figure S17. ^{13}C NMR spectrum of compound **3** in $CDCl_3$ (150MHz) 11

Figure S18. COSY spectrum of compound **3** in $CDCl_3$ (600 MHz) 11

Figure S19. HSQC spectrum of compound **3** in $CDCl_3$ (600 MHz) 12

Figure S20. HMBC spectrum of compound **3** in $CDCl_3$ (600 MHz) 12

Figure S21. ROESY spectrum of compound **3** in $CDCl_3$ (600 MHz) 13

Figure S22. CD spectrum of compound **1** 13

Figure S23. CD spectrum of compound **2** 14

Figure S24.CD spectrum of compound **3** 14

Figure S25. LC-HRMS finger-prints by ion extraction of co-culture, single strain, liquid medium of *C. globosum* fermentation products, red ginseng and compound (**2**, **3**) 15

Figure S26. Cytotoxicity of compounds **1**, **2** by MTS method 16

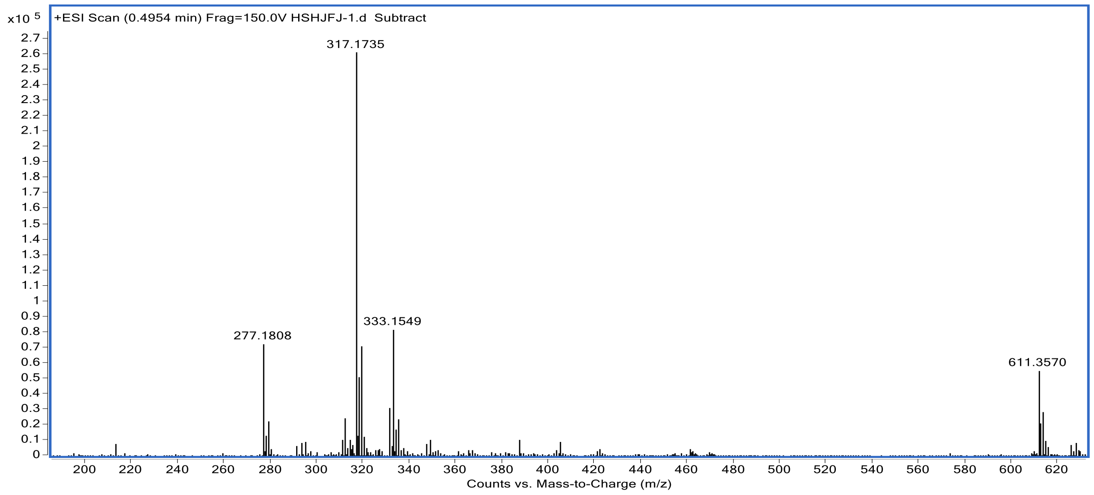

Figure S1. HRESIMS spectrum of compound **1**

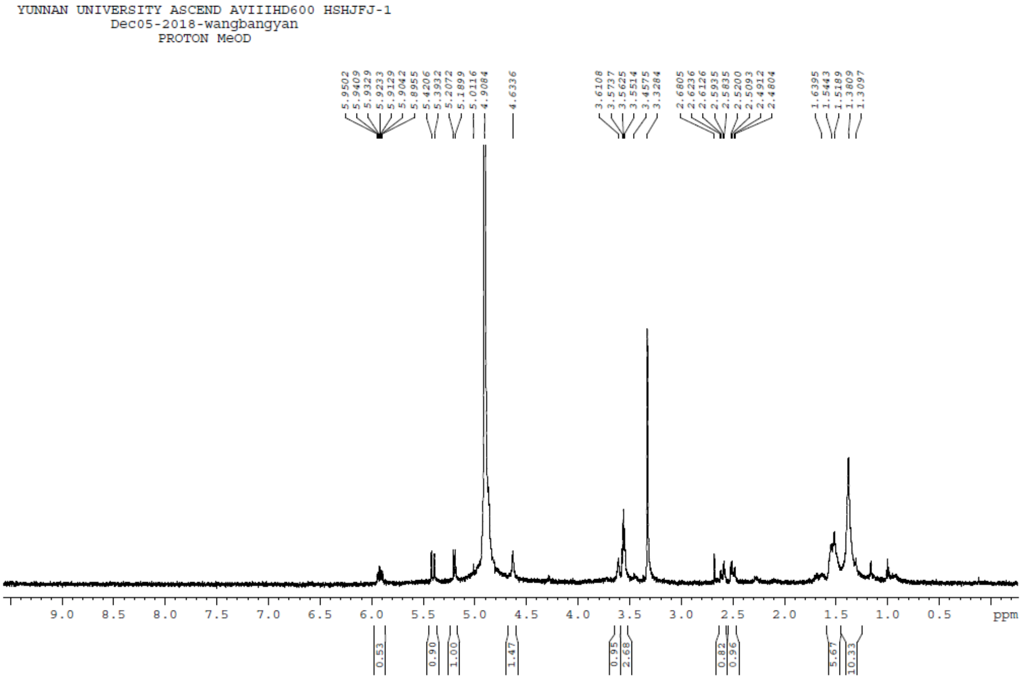

Figure S2. ^{1}H NMR spectrum of compound **1** in MeOD (600MHz)

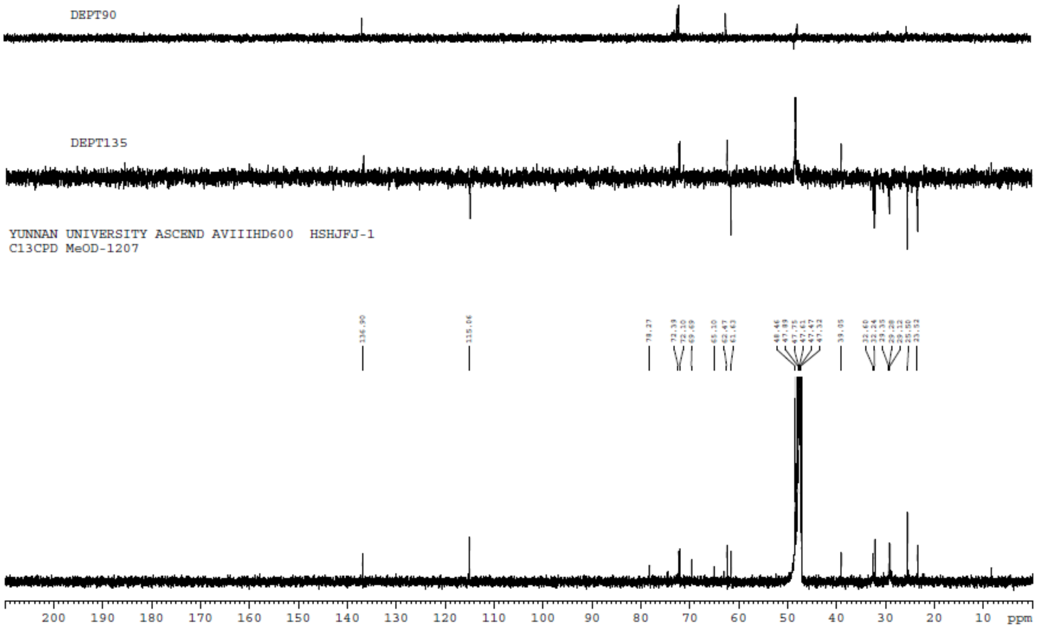

Figure S3. ^{13}C NMR spectrum of compound **1** in MeOD (150MHz)

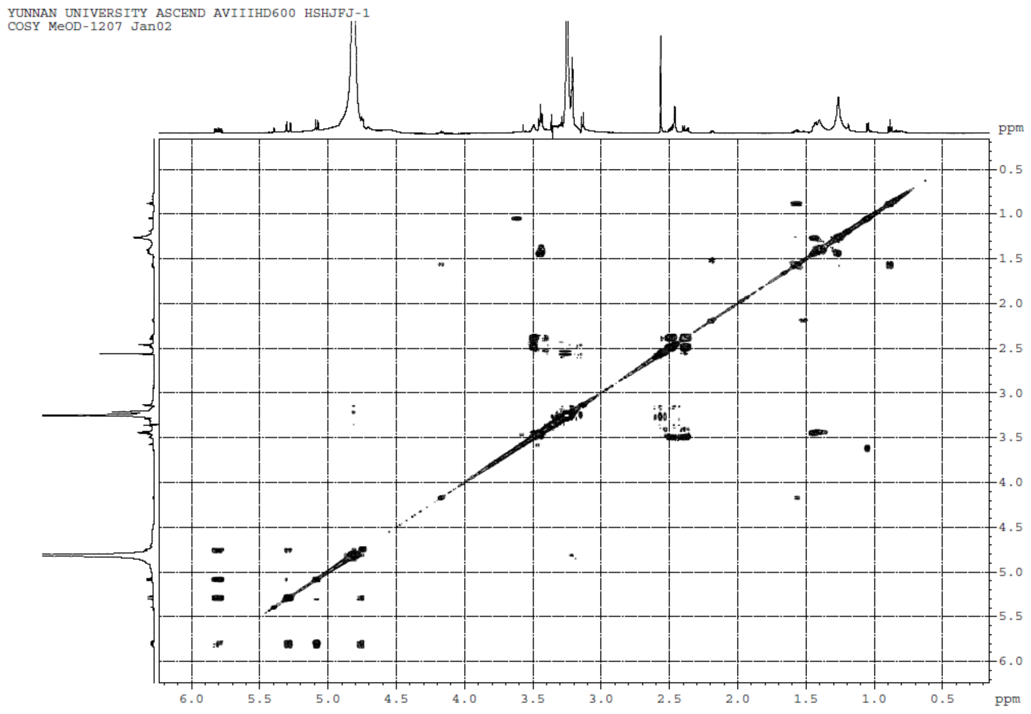

Figure S4. COSY spectrum of compound **1** in MeOD (600 MHz)

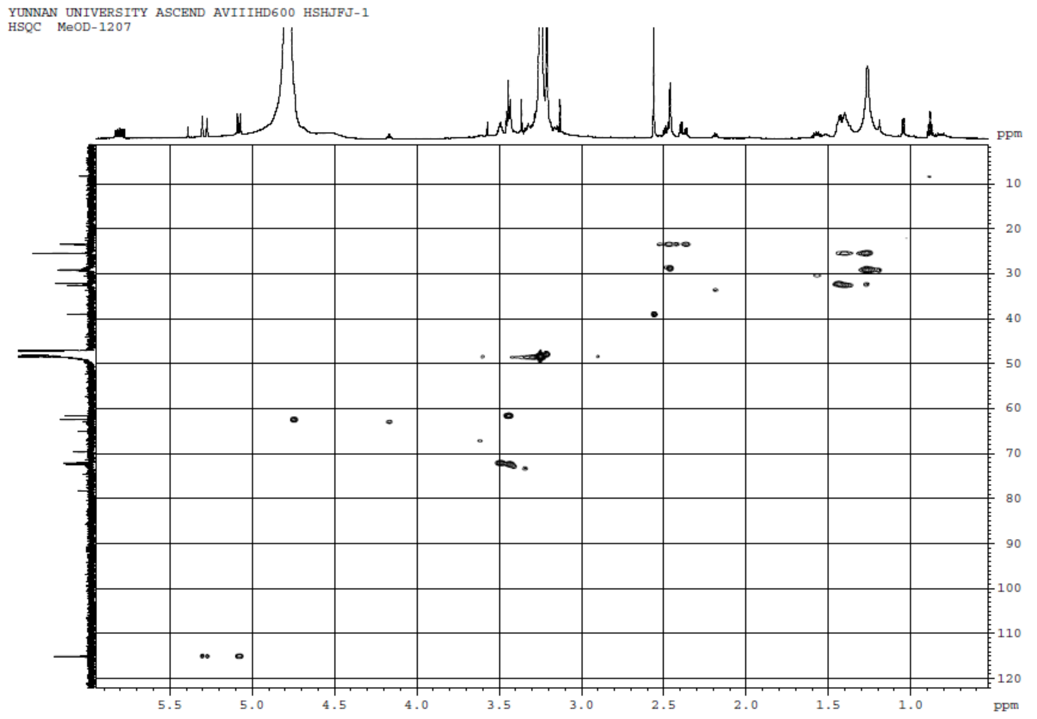

Figure S5. HSQC spectrum of compound **1** in MeOD (600 MHz)

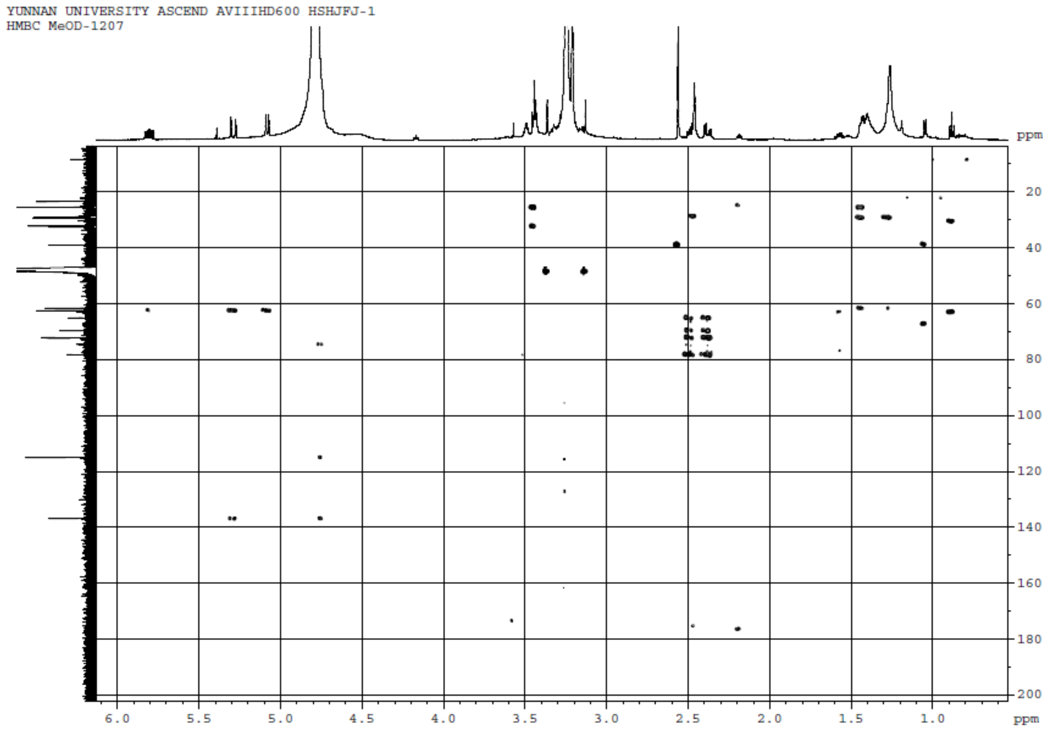

Figure S6. HMBC spectrum of compound **1** in $CDCl_3$ (500 MHz)

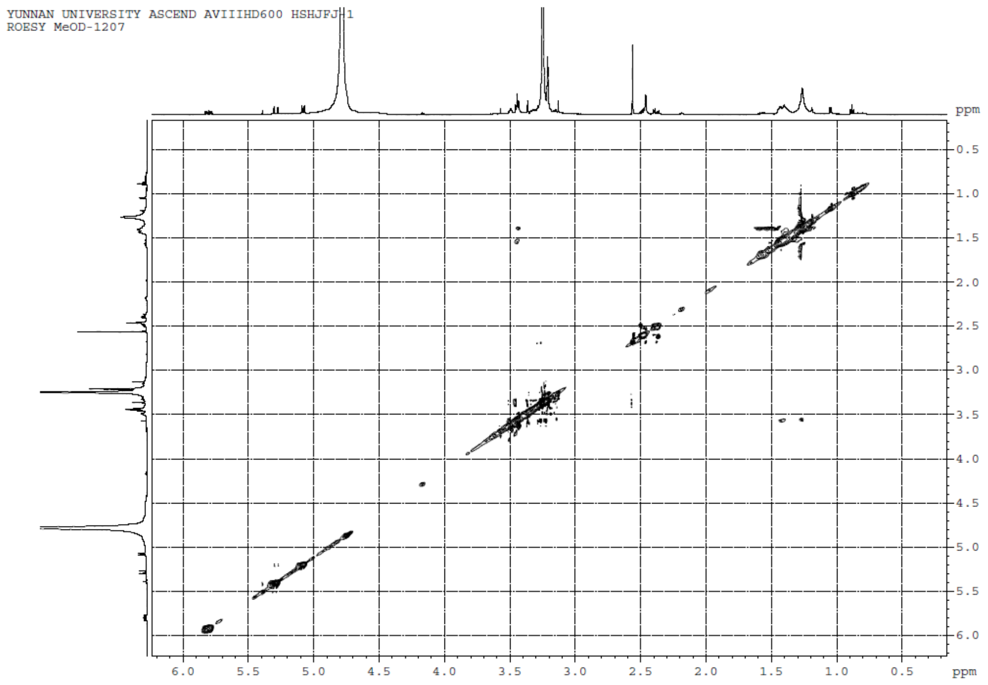

Figure S7. ROESY spectrum of compound **1** in $CDCl_3$ (500 MHz)

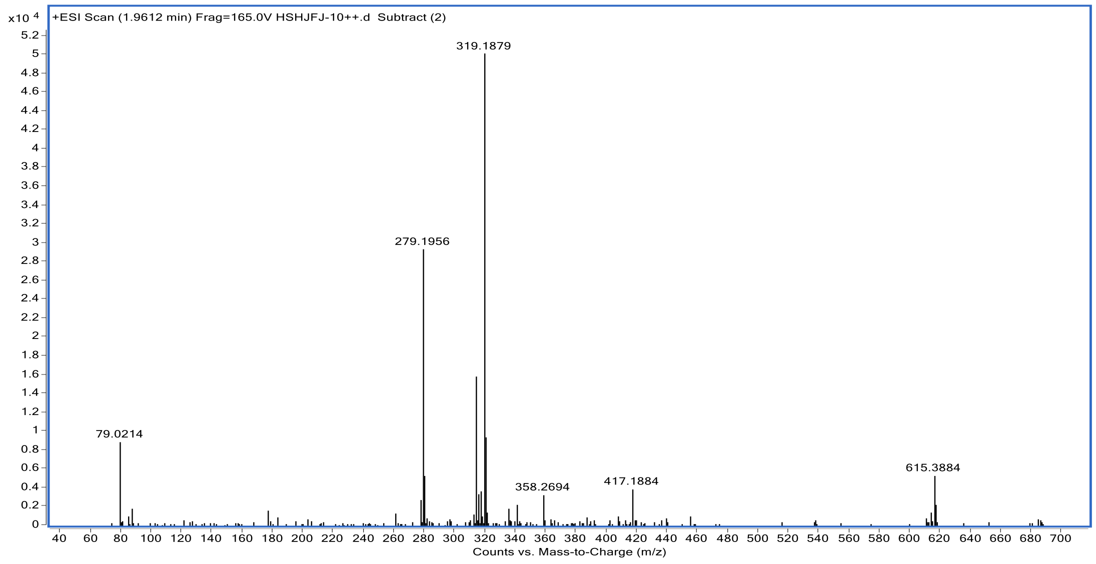

Figure S8. HRESIMS spectrum of compound **2**

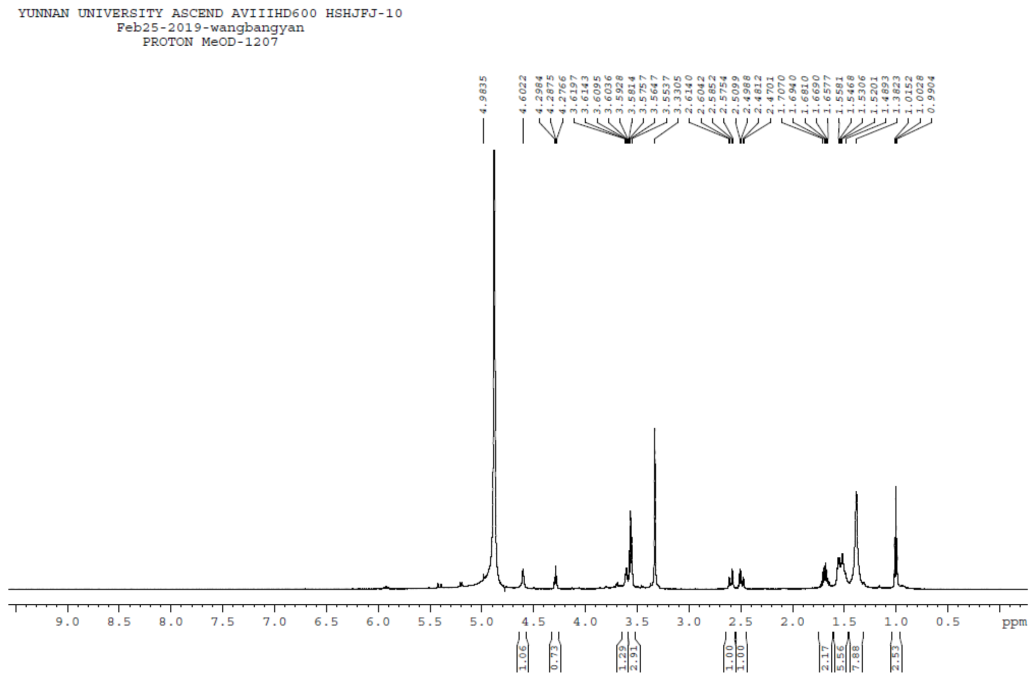

Figure S9. ^{1}H NMR spectrum of compound **2** in MeOD (600MHz)

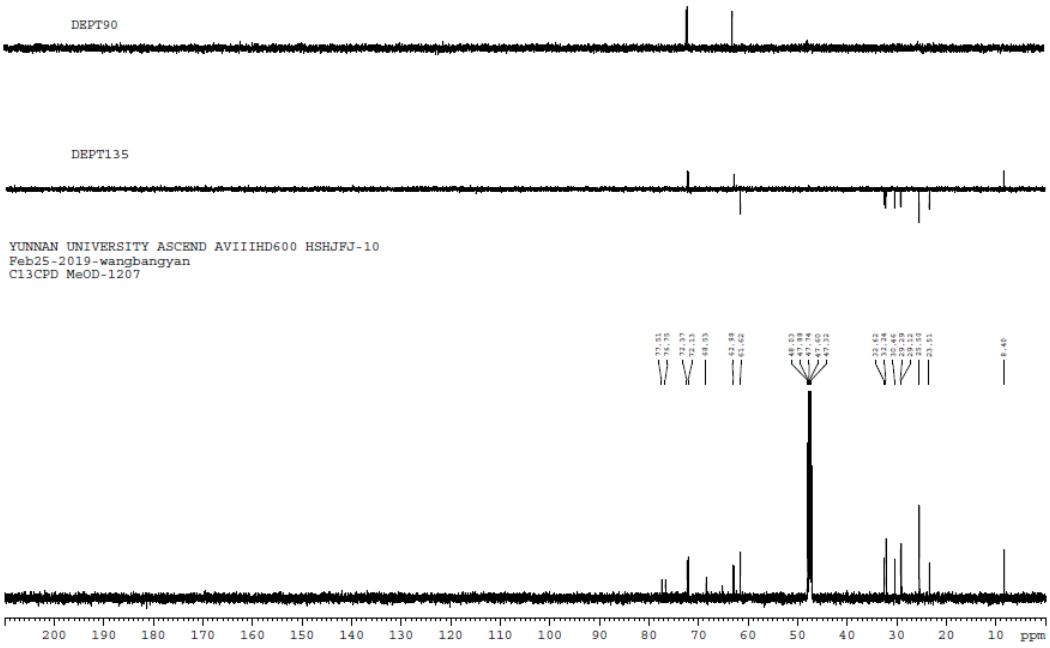

Figure S10. ^{13}C NMR spectrum of compound **2** in MeOD (150MHz)

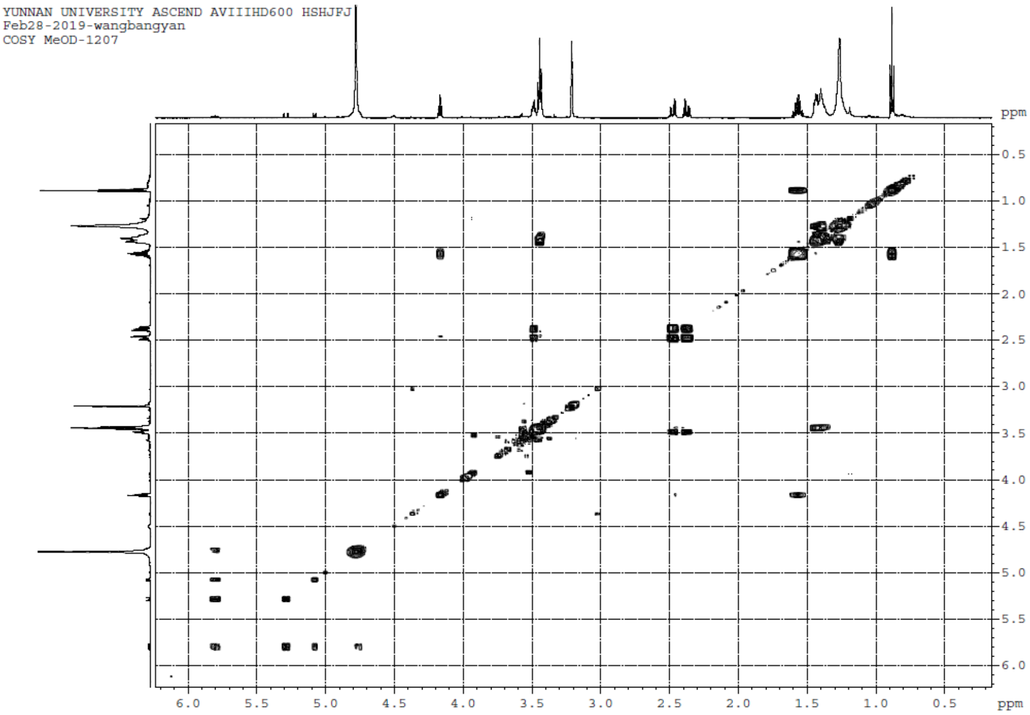

Figure S11.COSY spectrum of compound **2** in MeOD (600 MHz)

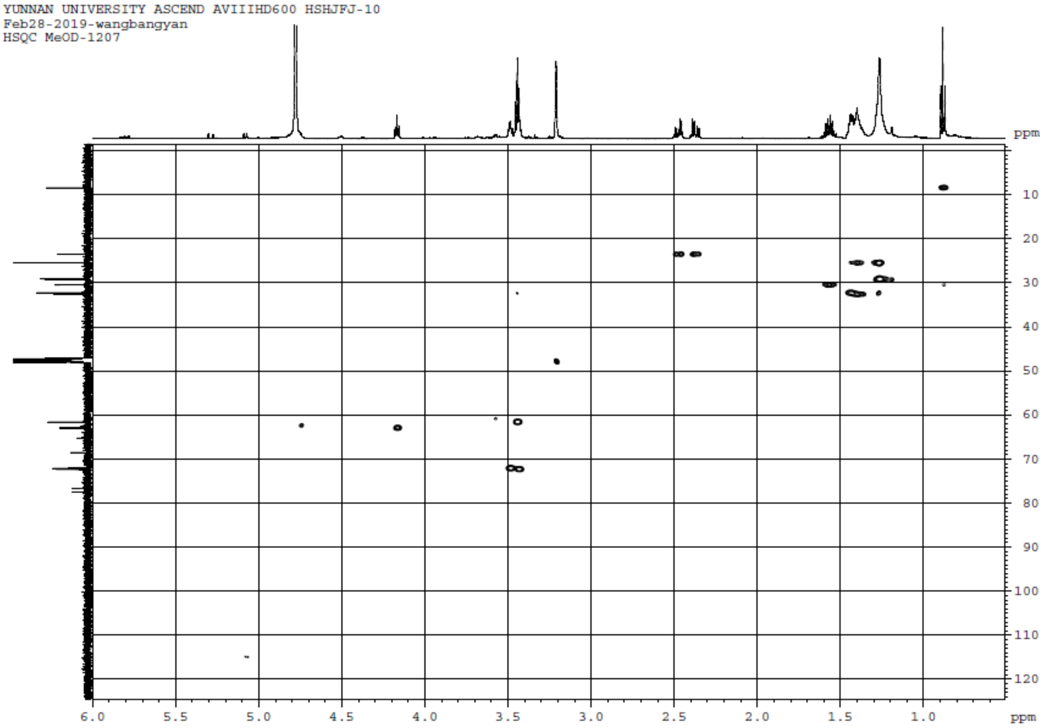

Figure S12. HSQC spectrum of compound **2** in MeOD (600 MHz)

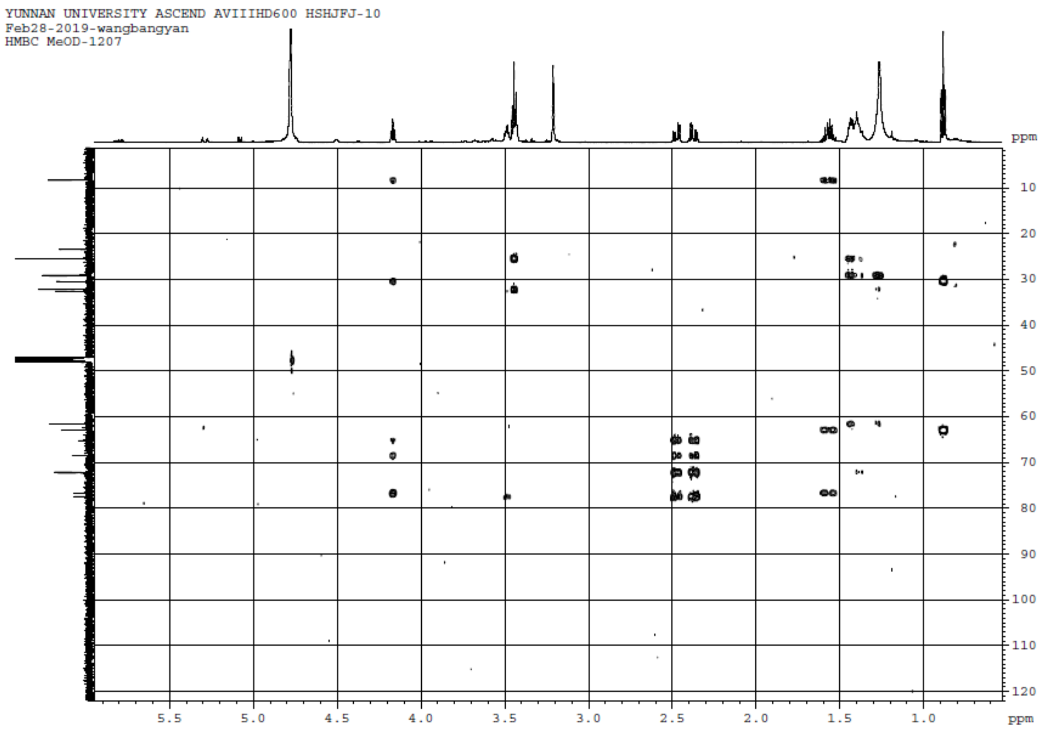

Figure S13. HMBC spectrum of compound **2** in $CDCl_3$ (600 MHz)

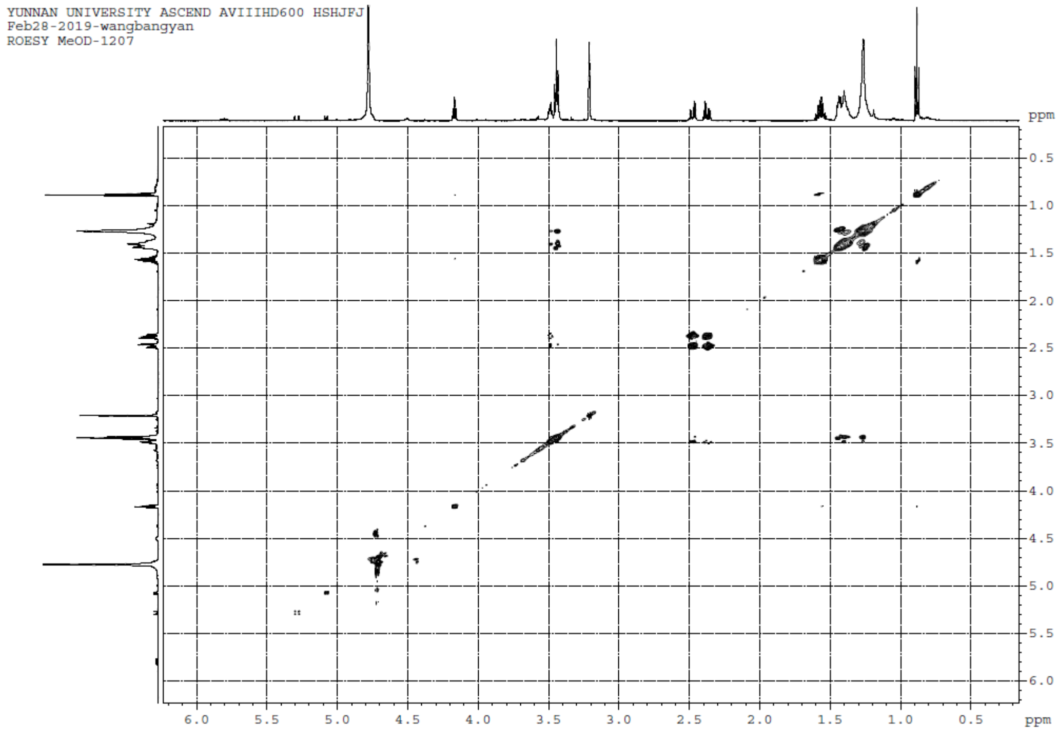

Figure S14. ROESY spectrum of compound **2** in MeOD (600 MHz)

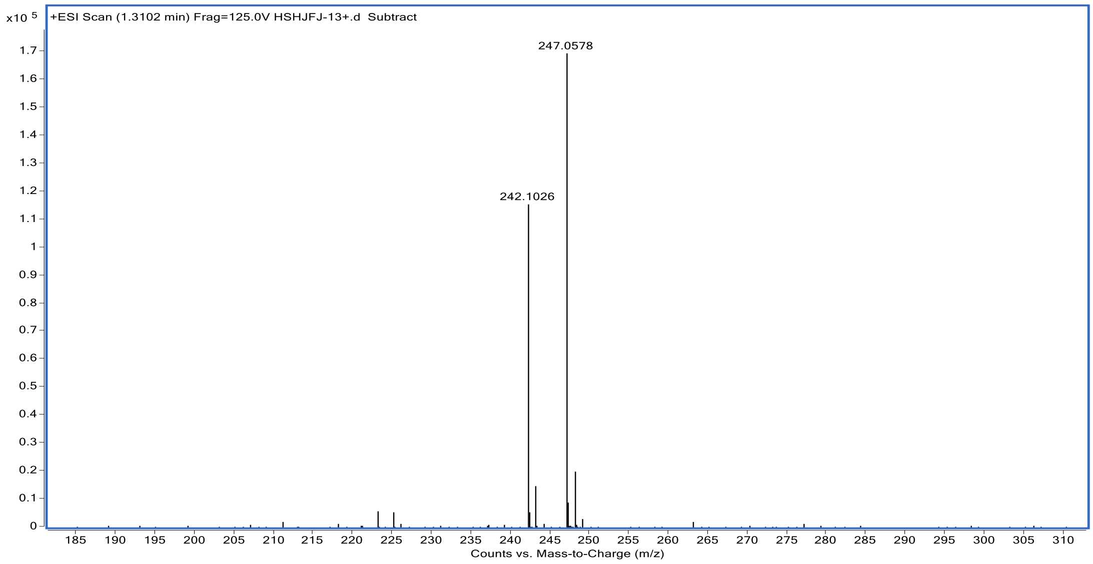

Figure S15. HRESIMS spectrum of compound **3**

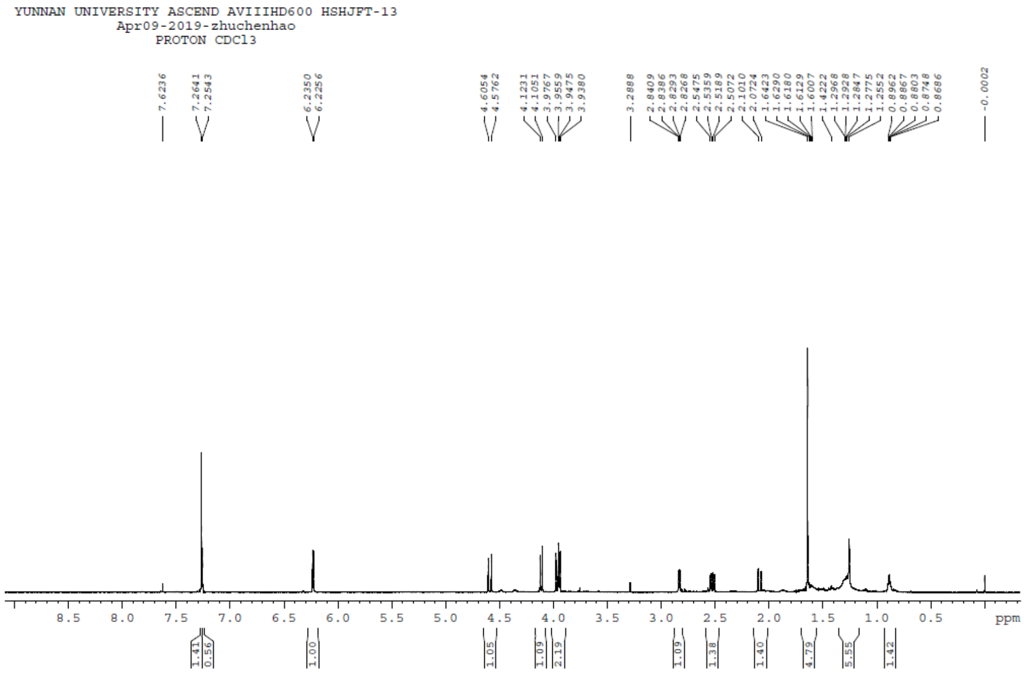

Figure S16. ^{1}H NMR spectrum of compound **3** in $CDCl_3$ (600MHz)

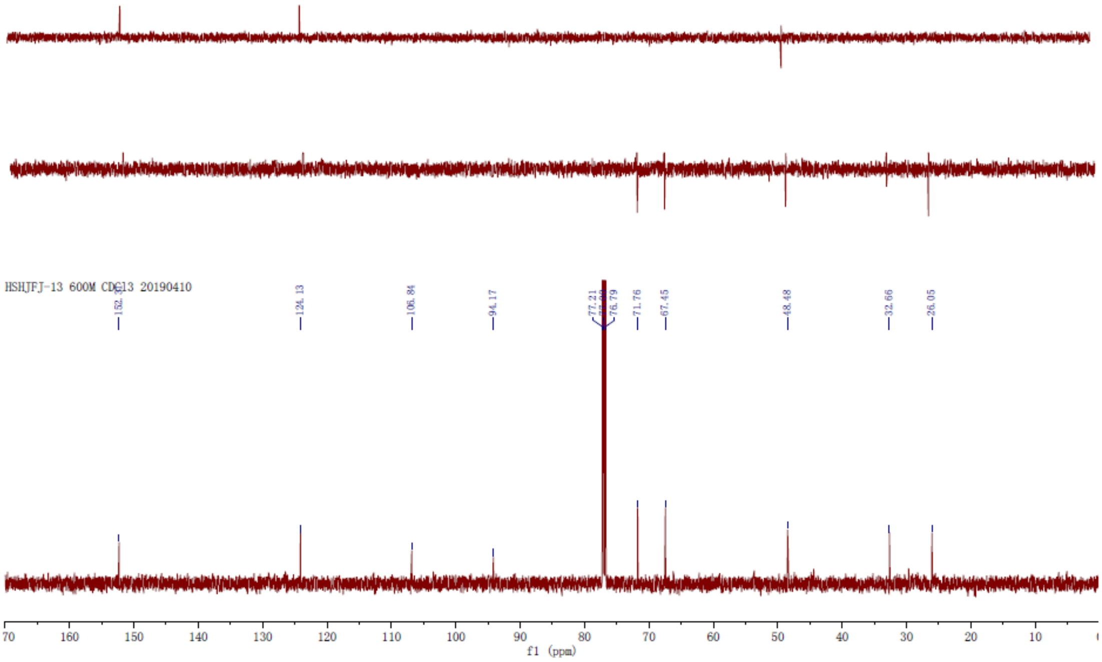

Figure S17. ^{13}C NMR spectrum of compound **3** in $CDCl_3$ (150MHz)

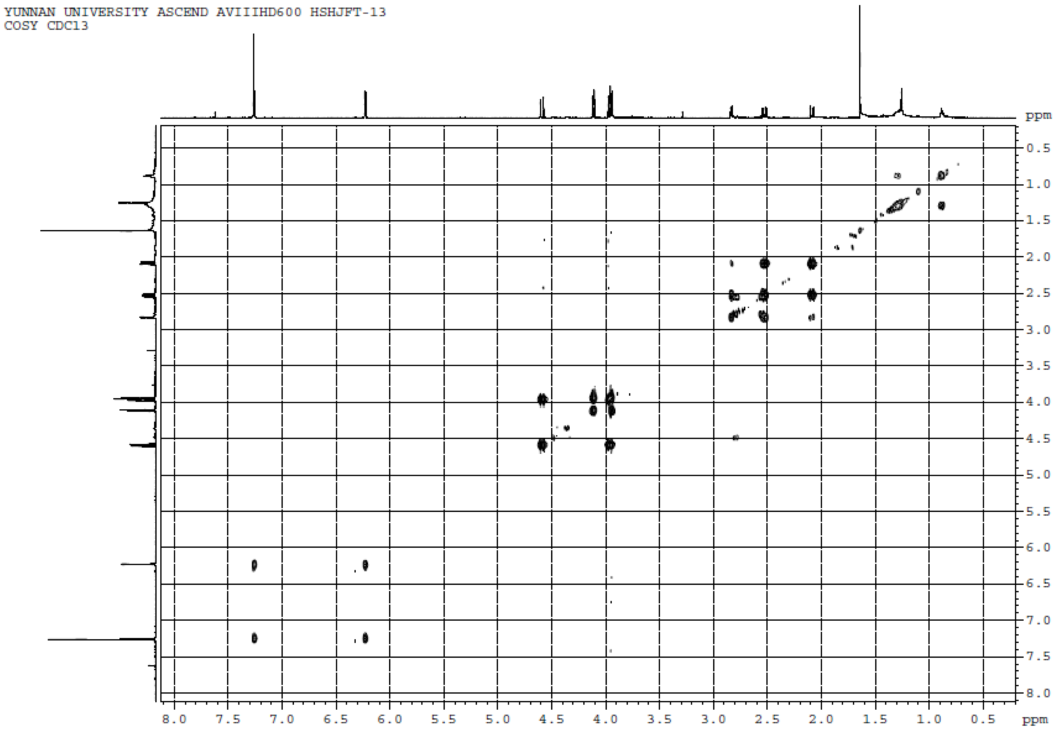

Figure S18. COSY spectrum of compound **3** in $CDCl_3$ (600 MHz)

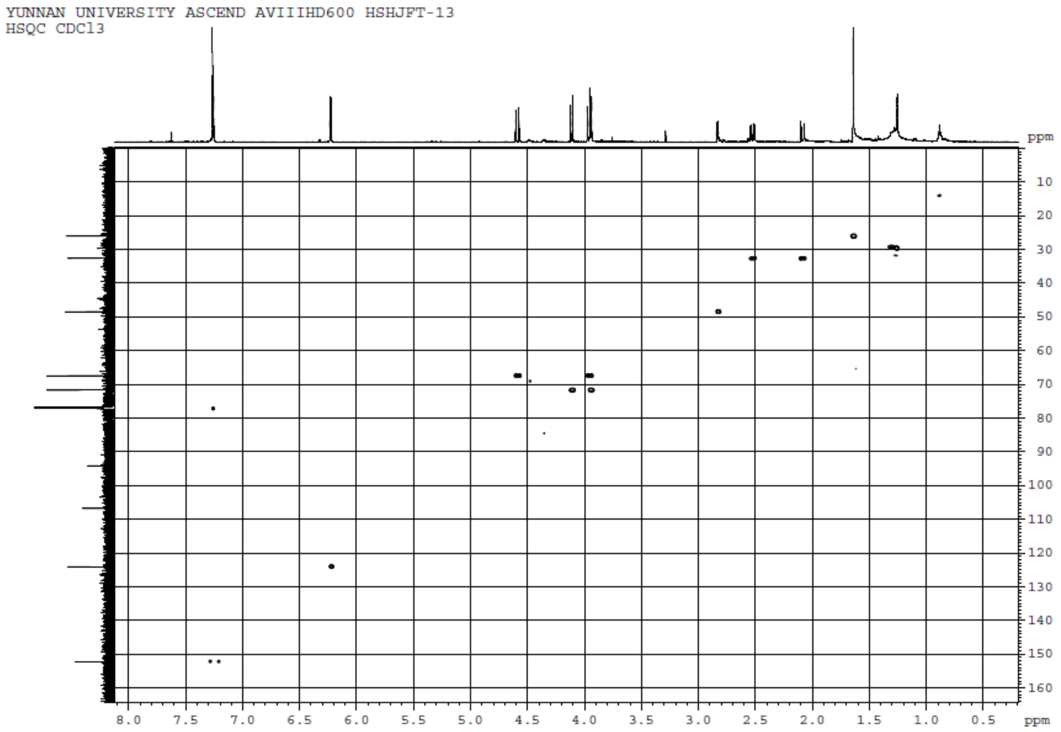

Figure S19. HSQC spectrum of compound **3** in $CDCl_3$ (600 MHz)

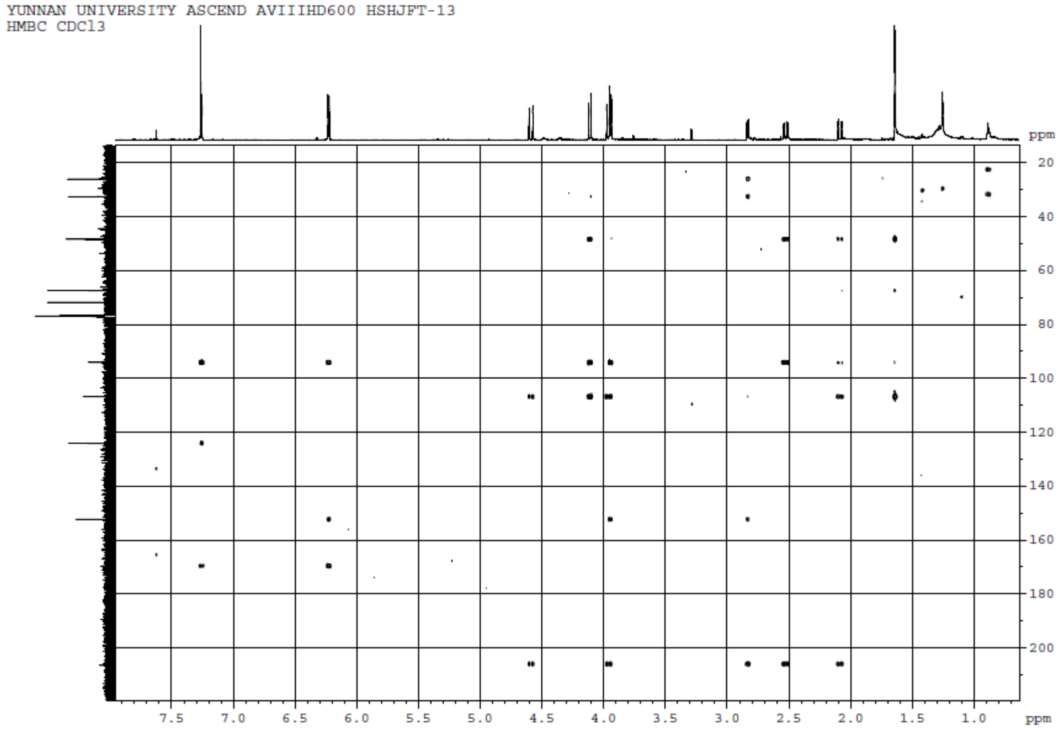

Figure S20. HMBC spectrum of compound **3** in $CDCl_3$ (600 MHz)

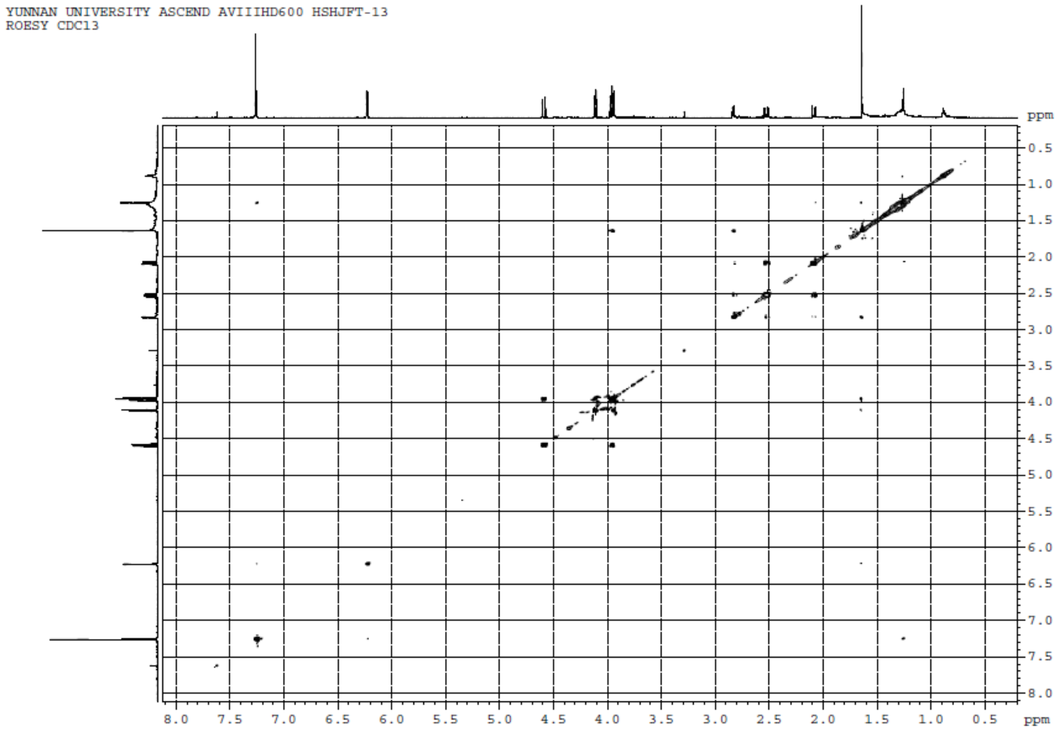

Figure S21. ROESY spectrum of compound **3** in $CDCl_3$ (600 MHz)

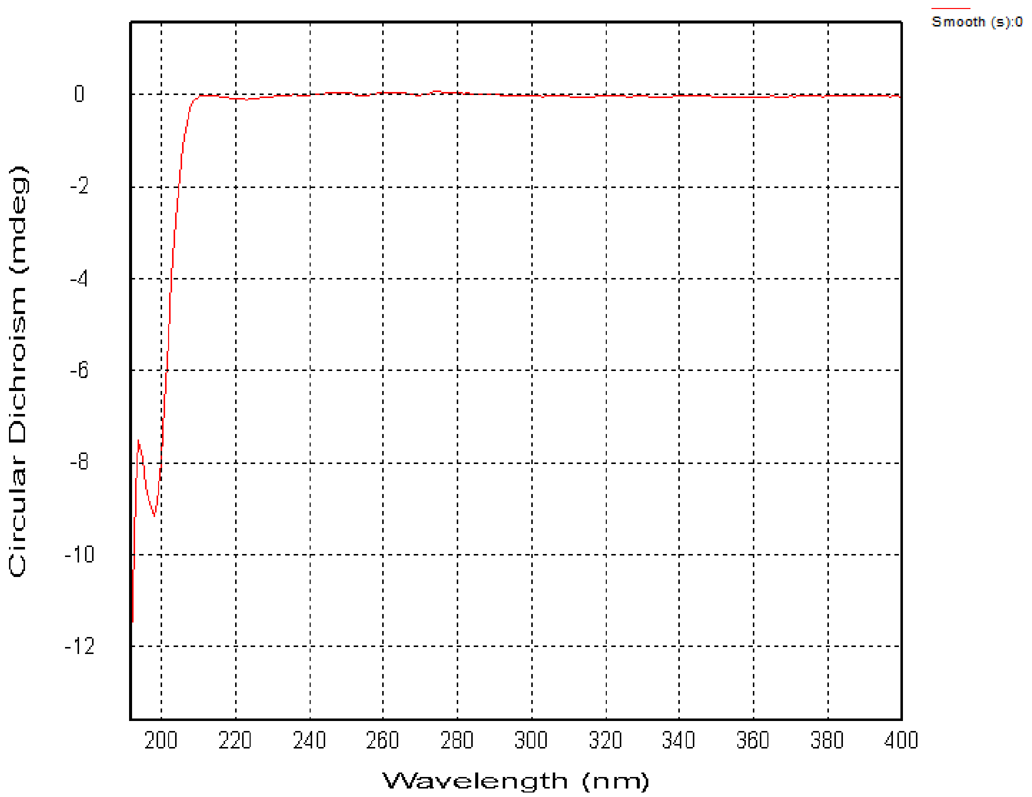

Figure S22. CD spectrum of compound **1**

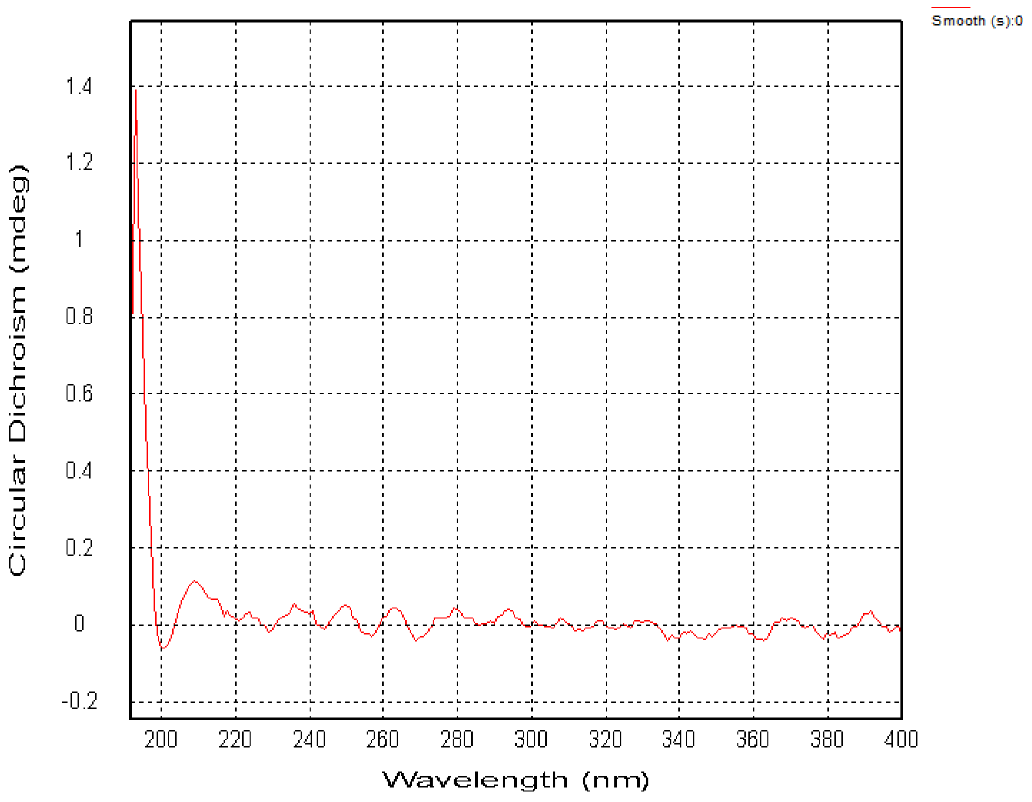

Figure S23. CD spectrum of compound **2**

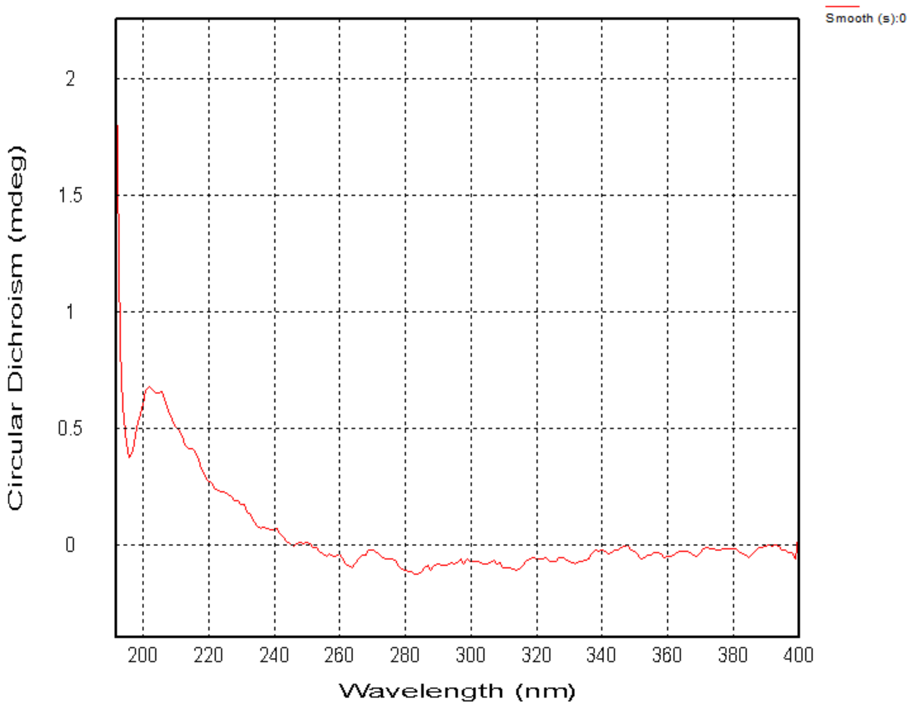

Figure S24. CD spectrum of compound **3**

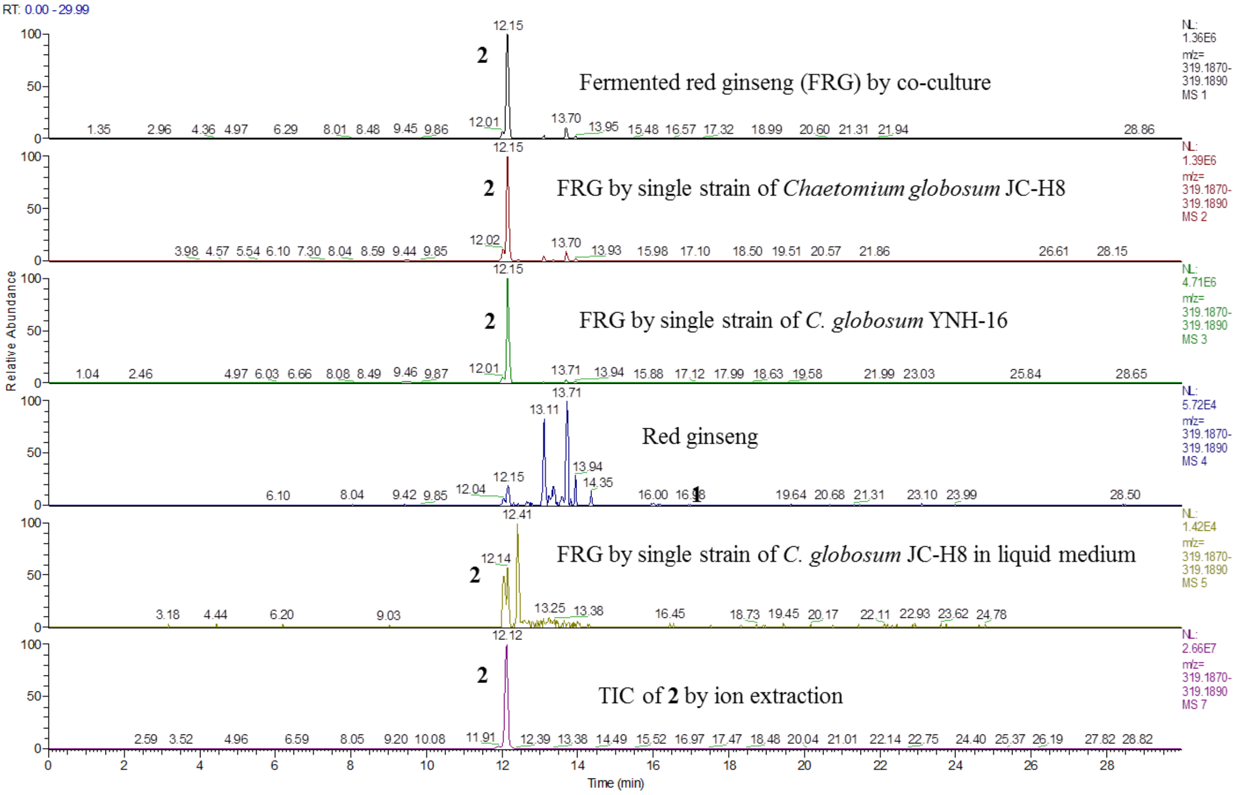

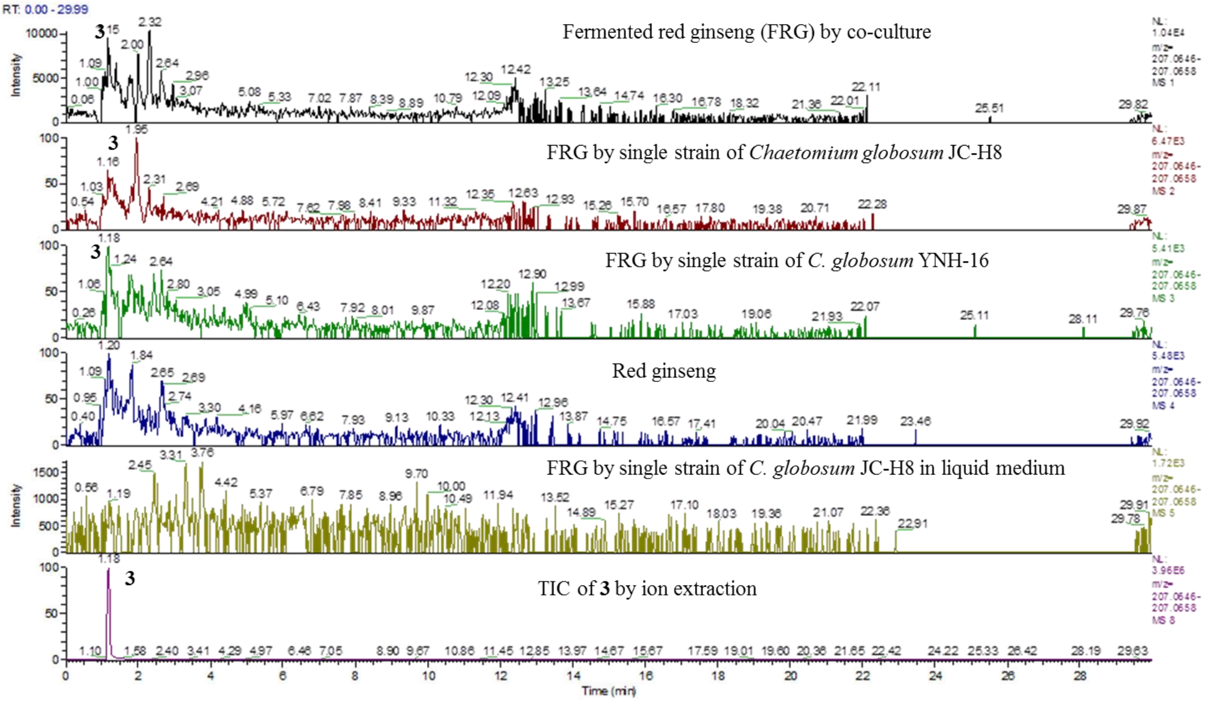

Figure S25. LC-HRMS finger-prints by ion extraction of co-culture, single strain, liquid medium of *C. globosum* fermentation products, red ginseng and compounds (**2**, **3**).

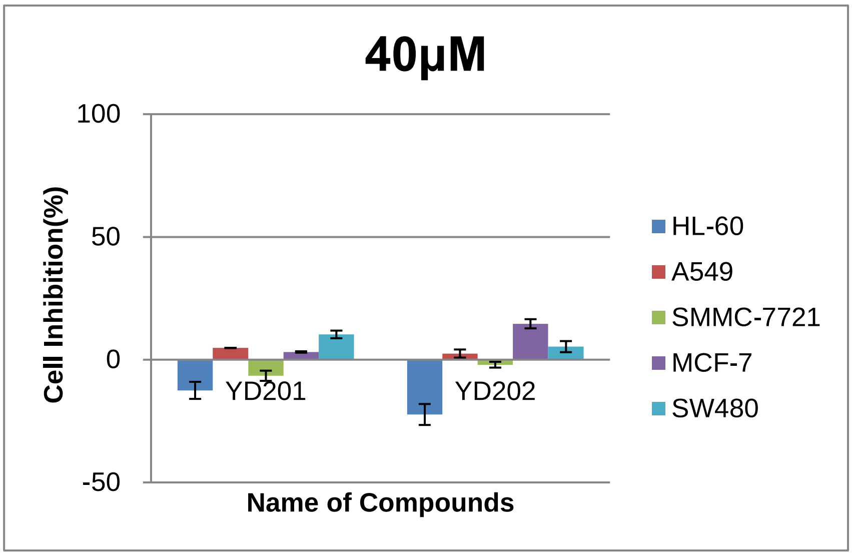

YD201=compound **1** YD202=compound **2**

Figure S26. Cytotoxicity of compounds **1**, **2** by MTS method
